# Advances in diagnosis and treatment of perimenopausal syndrome

**DOI:** 10.1515/biol-2022-0754

**Published:** 2023-12-22

**Authors:** Wanying Chen, Mengjuan Chen, Huimin Tang, Weiwei Wei, Panqiu Shao, Shulan Dou, Jia Wu, Bingying Lu, Ruxia Shi, Jiming Chen

**Affiliations:** Department of Gynecology, The Affiliated Changzhou No. 2 People’s Hospital of Nanjing Medical University, Changzhou, Jiangsu, 213003, PR China; Zhoukou Maternal and Child Health Hospital, Zhoukou, Henan, 466000, PR China

**Keywords:** perimenopausal syndrome, hormone replacement therapy, low dose, efficacy

## Abstract

With the development and progress of society, people’s average life expectancy has increased, and relevant literature reports that the number of postmenopausal women in China continues to increase. With lifespans extended, the transition period and post-menopause period have become the longest essential period in every woman’s life. The life quality of women troubled by perimenopausal syndrome has been significantly reduced, which also places a burden on families and society. It is well known that hormone replacement therapy plays a vital role in improving women’s menopause-related symptoms and is the most effective medical measure. With research ongoing into the treatment of menopausal symptoms in different patients, dose size, treatment duration, and medication regimens for hormones are still hot topics of discussion. This article reviews the definition, clinical diagnosis, staging, clinical manifestations, and treatment of menopause and explores the current diagnosis and treatment scenarios of perimenopausal syndrome.

Menopause is a life stage that every woman must go through. According to statistics, China had 167 million postmenopausal women in 2011, and it may reach 280 million by 2030. With life expectancies extended, more than one-third of a woman’s lifetime is in the postmenopausal stage. Therefore, it is conceivable that menopause-related health management and prevention and control of perimenopausal diseases have become important public health issues, which clinicians, scientists, and wider society should pay attention to [1]. Ovarian function failure is the leading cause of menopause. With the decline of ovarian function, the hypothalamus–pituitary–ovarian axis in women’s bodies becomes out of balance, resulting in related symptoms. This series of physical and psychological health problems is called perimenopausal syndrome and includes both short-term and long-term symptoms. Prominent symptoms include hot flashes, sweating, palpitations, paresthesia, irritability, depression, etc.

## 1 Clinical diagnosis and staging of menopause

**Figure 1:**
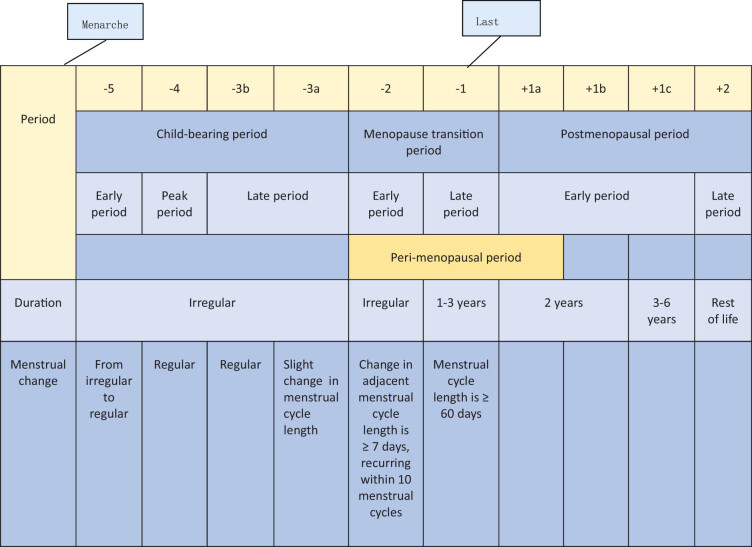
Stages of female reproductive system aging.

The clinical definition of menopause refers to the permanent cessation of a woman’s monthly menstruation. That is, a woman aged over 40 years who has no menstrual cramps within 12 months of their last menstrual period can be diagnosed as experiencing menopause once pregnancy and other related diseases have been ruled out. The essence of menopause does not simply refer to the presence or absence of menstruation but refers to the failure of female ovarian function, meaning that the female reproductive system is in a state of functional “exhaustion.” With the decline in ovarian function, the estrogen in the human body will also fluctuate and decline, and women will often have a series of physical and mental symptoms. The Stages of Reproductive Aging Workshop + 10 was announced in 2011 (Figure 1). It is currently a worldwide recognized staging benchmark for female reproductive aging [2]. The arrival of menarche marks the start of the female reproductive period. The duration of this lifecycle is variable. As age increases, the ovarian endocrine axis matures, and the menstrual cycle gradually turns from irregular to regular, including an early period (−5), peak period (−4), and late period (−3). In the latter period, women’s fertility has begun to decline, and the period can be further divided into two subperiods −3b and −3a. In the −3b subperiod, women’s menstruation can remain regular, and in the −3a subperiod, women’s menstrual cycle begins to show subtle changes, especially cycle shortening. Women’s menopausal transition period can be divided into early (−2) and late (−1) periods, and the change in the menstrual cycle is the main evaluation criteria. The menstrual cycle length change (that is, menstrual disorder) is a sign of the early transition period for menopausal women. Specifically, it refers to a length change in 2 or more adjacent menstrual cycles that is ≥7 days in 10 menstrual cycles. Menstrual cycle extension being ≥60 days is a sign of late menopause transition. Postmenopause includes early (+1) and late (+2) periods. The early period can be divided into three sub-periods, namely +1a, +1b, and +1c, of which the +1a sub-period refers to 1 year after the last menstrual period. The menopause transition period and the +1a sub-period are also known as perimenopause. Menopause can be clearly diagnosed only after the +1a sub-period ends. The +1b sub-period lasts for 1 year, the +1c sub-period lasts for 3–6 years, and the +2 sub-period is the late postmenopause. Menopause is also categorized into natural menopause and artificial menopause after intervention. It means the reduction of women’s ovarian function. If a woman receives an artificial hysterectomy but her ovarian function is normal, she does not belong to the menopause category. Patients with perimenopausal syndrome may suffer from a series of other systemic symptoms, and medical workers need to rule out related organic diseases, such as thyroid dysfunction, cardiovascular and cerebrovascular diseases, mental diseases, and bone and joint system diseases. The laboratory manifestations of female ovarian function decline generally require the detection of sex hormone levels. When the anti-Müllerian hormone (AMH) is less than 0.2 ng/mL, the follicle-stimulating hormone is >40 U/L, and the estrogen is <10–20 pg/mL, it indicates that the woman is about to go into menopause, and AMH is generally undetectable in the blood of postmenopausal women.

## 2 Clinical manifestations of perimenopausal syndrome

### 2.1 Menstrual disorders

Irregular menstrual cycles, long menstrual periods and increased or decreased menstrual blood volumes are the main menstrual-related manifestations among women. Other studies have shown that 82% of perimenopausal women have problems related to amenorrhea, a prolonged menstrual cycle, and/or oligomenorrhea, of which 18% have symptoms of increased menstrual blood volumes, menostaxis, or short menstrual cycles. It was found that 19% of the patients with short menstrual cycles had malignant changes such as histological precancerous lesions. Moreover, abnormal uterine bleeding during perimenopause is primarily due to anovulatory dysfunctionality, often manifested in weeks or months of menopausal symptoms, followed by sudden massive vaginal bleeding or irregular uterine bleeding, while anemia, infection, and shock are more serious complications [3].

### 2.2 Vasomotor symptoms (VMS)

Hot flashes are the main manifestation of VMS and a characteristic symptom of ovarian hypofunction in women. The main pathogenesis is that the fluctuation of estrogen and other endocrine hormones in the female body leads to vasomotor dysfunction, resulting in hot flashes. The incidence of hot flashes in menopausal women may reach as high as 75%, and the most common manifestation is redness of skin on the face, chest, and neck, followed by sweating, which usually lasts for minutes and can occur several times a day. In more severe cases, the symptoms can occur dozens or more times a day [4].

### 2.3 Symptoms of autonomic disorders

Headache, palpitations, insomnia, dizziness, and tinnitus are common symptoms of autonomic disorders. Common neuropsychiatric symptoms, such as agitation, irritability, depression or anxiety, and a lack of self-control, may be accompanied by hypomnesis. According to relevant studies and statistics, the incidence of neurological symptoms in menopausal women is as high as 58%. The specific manifestations reported are depression (accounting for 78%), irritability (accounting for 72%), apathy (accounting for 65%), insomnia (accounting for 52%), and headache (accounting for 35%) [5,6].

### 2.4 Long-term symptoms

**Figure 2:**
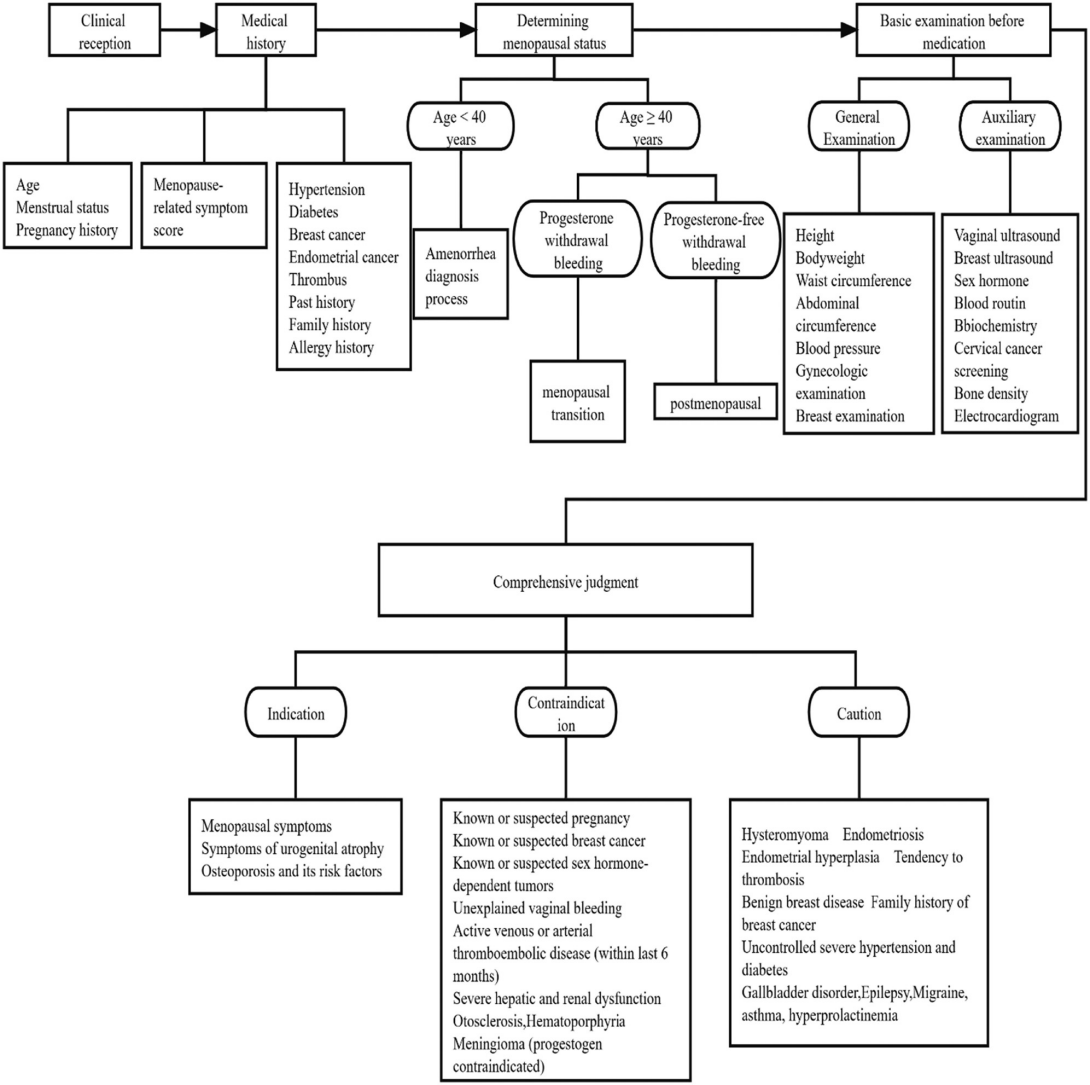
Menopause clinic flowchart.

Genitourinary syndrome of menopause (GSM) is characterized by vaginal dryness, dyspareunia, and recurrent vaginal infections, as well as recurrent urinary tract infections such as dysuria, odynuria, and urgent urination. Relevant literature shows that more than 50% of menopausal women will experience symptoms related to the genitourinary system, largely due to the reduction of postmenopausal hormone levels, which leads to different degrees of atrophic changes in the human reproductive system. This includes atrophy of the labia minora in postmenopausal women, thinning and less flexibility of mucous membrane at the vaginal wall, vaginal dryness, dyspareunia, and persistent vaginal infections caused by varying degrees of cervix and uterine atrophy, as well as endometrial inflammation, pyometra, and more [7]. Osteoporosis is due to the lack of estrogen in postmenopausal women, leading to an increase in bone absorption and massive loss of bone mass, which in turn causes a significant decrease in bone content and bone strength and gradually develops into osteoporosis [8]. Some studies have shown that estrogen can effectively reduce the incidence and mortality of cardiovascular- and cerebrovascular-related diseases. The reduction of estrogen in women in menopause reduces the protective effect on the cerebrovascular system, resulting in functional degeneration of the cardiovascular and cerebrovascular systems and increasing the risk of cardiovascular and cerebrovascular diseases in postmenopausal women, such as coronary heart disease and arteriosclerosis [5,9] (Figure 2).

## 3 General treatment of perimenopausal syndrome

After women enter the perimenopausal period, their basal metabolic rate will decrease. Without proper management, weight will increase. Some studies have shown that weight gain is a risk factor for knee joint disease, as the occurrence of knee joint disease in overweight and obese people is significantly higher than that in people of a normal weight [10]. Weight control through diet management can reduce or avoid bone and joint damage, and control of coffee, strong tea, and alcohol intake can also reduce bone loss. According to the Global Burden of Disease Study, unreasonable diet is the most significant cause of disease and death among Chinese residents. Among the causes of death among Chinese residents in 2017, 3.1 million deaths can be attributed to their unreasonable diet [11]. Therefore, healthy diet management is critical for perimenopausal patients. The Chinese Nutrition Society recommends that Chinese women in menopause consume whole grain fiber and ensure enough intake of fresh fruits and vegetables every day. Specifically, they should eat fish-based food at least twice a week, reduce sugar and oil intake, and limit salt and alcohol intake. The daily intake of sugar should be ≤50 g, salt should be ≤6 g/day, oil should be 25–30 g, and alcohol content should be ≤15 g. Smoking is not recommended, and sufficient water should be consumed, which is recommended to be 1,500–1,700 ml per day. Regular aerobic exercise is recommended every day to reach a total of 150 min per week, and resistance exercise should be carried out twice to thrice a week to increase muscle mass and strength in the body. The 2020 WHO guidelines on physical activity and sedentary behavior also mentioned that adults should set a target of 150–300 min of moderate-intensity and 75–150 min of vigorous-intensity physical activities [12]. A total of 6,000 steps of physical activity on average per day is recommended to enhance the overall health of menopausal patients. In terms of diet, the literature shows that the risk of all-cause mortality decreases by up to 12% among people with a high intake of whole grains, and there is a clear dose–response relationship between the two. Soy isoflavones, the main component of soybeans, can reduce bone absorption, increase bone density, and prevent osteoporosis. High doses (>90 mg/day) are beneficial to bone density of hip joints and lumbar spine. The intake of soy and its products can also reduce the risk of breast cancer. Every 10 mg/day increase of soy isoflavones can reduce the risk of breast cancer by 3%. More broadly, it was found that the consumption of soy and its products (soy intake >1.62 g/day, or tofu intake ≥14.4 g/day, or soy isoflavones at 26.3 mg/day) can greatly reduce the risk of breast cancer in women (especially postmenopausal women).

## 4 Hormone therapy for perimenopausal syndrome

Menopausal hormone therapy (MHT) is the most common and important treatment method for postmenopausal women. It refers to the artificial supplementation of estrogen and progesterone to improve a series of menopausal syndromes caused by low levels of estrogen and progesterone in the body. According to the Hormone Therapy Position Statement issued by the North American Menopause Society (NAMS) in 2017, supplemental hormone therapy is currently the most effective medical measure for the treatment of VMS and GSM [13]. Studies have shown that oral hormone therapy can significantly reduce the incidence of VMS during menopause and reduce their severity to significantly improve the overall quality of life for menopausal women [14,15]. Studies have shown that MHT can also reduce bone destruction by inhibiting the activity of osteoclasts to control the osteopenia degree in postmenopausal women. Women who start MHT treatment right before or after menopause can achieve the effect of primary fracture prevention [16,17]. The International Menopause Society proposed in 2017 the concept of “time window” [18]. The Global Consensus Statement on Menopausal Hormone Therapy in 2013 clearly stated that menopausal women younger than 60 years old or within 10 years of menopause are in a “time window” period, during which hormone therapy can generate the highest benefit, with relatively low risks of treatment [19].

### 4.1 Commonly used drugs for hormone therapy

#### 4.1.1 Estrogens

Relevant studies recommend the use of natural estrogens, including 17β-estradiol, conjugated estrogens, and estradiol valerate. Preparations that can be absorbed through the skin include estradiol gel, estradiol patches including estradiol hemihydrate patches, and vaginal administration including conjugated estrogen ointment, estriol ointment, and prostalene vaginal capsules. Non-oral hormone replacement therapy (transdermal therapeutic system) is an important step forward in HRT in recent years, especially for postmenopausal women with chronic liver and gallbladder diseases, gastrointestinal tract diseases, and others who cannot tolerate oral administration. Non-oral estrogen supplementation perfectly avoids the first-pass effect of the human liver, with smaller stimulation to the liver and a smaller impact on metabolism. Therefore, non-oral hormones as complementary treatment regimens are advantageous as they can reduce the risk of cardiovascular and cerebrovascular diseases and venous thrombosis.

#### 4.1.2 Progesterone

Micronized progesterone is a natural progesterone, and dydrogesterone is a reversal progesterone derivative, and also the drug most resemblant to natural progesterone. Relevant treatment guidelines also recommend that menopausal women use natural progesterone or the progestogen that is the closest to natural progesterone.

#### 4.1.3 Compound preparations of estrogen and progesterone

The sequential preparations of estrogen and progesterone include estradiol/estradiol-dydrogesterone tablets and estradiol valerate/estradiol valerate and cyproterone acetate tablets. The continuous combined preparation of estrogen and progesterone is estradiol/drospirenone tablets.

#### 4.1.4 Tibolone

7-Methyl-norethisterone is the main active ingredient of tibolone, which is a selective modulator of estrogenic activity. In addition to having good effects on menopause-related symptoms such as those present in mood, skeletal system, and atrophic vaginitis, it will not stimulate endometrial hyperplasia and will not increase the density of female breast tissue and the incidence of breast distending pain. For postmenopausal women with a uterus, tibolone treatment does not require progesterone to antagonize endometrial proliferation. Tibolone metabolites contain androgenic related activities, which can also effectively ease mood and increase libido.

### 4.2 Commonly used treatment regimens for MHT

#### 4.2.1 Relevant treatment regimens using progesterone alone

It is primarily used in the early menopausal transition period. Their main purpose is to treat menstrual-related problems in the process of ovarian decline.

#### 4.2.2 Treatment regimens using estrogen alone

It is only for postmenopausal women undergoing hysterectomy,

#### 4.2.3 Combined use of estrogen and progesterone, including estrogen-progestin sequential regimens and estrogen-progestin combination regimens

##### 4.2.3.1 Sequential regimen

It is primarily used in women who have an intact uterus, are in perimenopause or postmenopause but still wish to maintain menstrual-like bleeding. The regimens can be divided into cycle sequential ones and continuous sequential ones. The cycle sequential regimen refers to the use of estradiol valerate tablets/estradiol cyproterone tablets belonging to the cycle sequential compound preparation, with 1 tablet per day for a total of 21 days, and a pause in drug dosing for 7 days before the next cycle starts. Continuous oral or transdermal estrogen administration for 21–25 days can also be chosen, with progesterone added during the last 10–14 days. After the drug dose is paused for 3–7 days, the next cycle of treatment starts. The continuous sequential regimen refers to continuous application of estrogen without a stop, with progesterone added during the last 10–14 days of each month. Withdrawal vaginal bleeding is expected. Relatively speaking, the continuous sequential regimen uses estrogen without interruption, which is more beneficial for the control of menopause-related symptoms.

##### 4.2.3.2 Continuous combination regimen

Primarily used for women who have an intact uterus and do not wish to have menstrual-like bleeding after menopause. In addition, for postoperative patients with endometriosis, the continuous combination regimen is generally recommended in the early stage to reduce the adverse effects of endometriosis on ovarian reserve. Daily continuous administration of estrogen (oral or transdermal) plus progestin can be chosen, or a combination preparation could be used, such as estradiol/drospirenone tablet, with 1 tablet per day. It is also administered continuously. The continuous combination regimen is easy to use, has a low rate of vaginal bleeding, and has good compliance.

### 4.3 Low-dose MHT

#### 4.3.1 Overview of low-dose MHT

The type and method of MHT medication and the choice of therapeutic dose should be comprehensively analyzed according to the patient’s situation (such as the patient’s expected effect of the treatment and relevant health examination results). The hormone supplementary treatment with the most benefit can be prioritized after analysis of the patient’s condition. Each patient’s situation is different, so individualized treatment should be conducted during the consultation process. The NAMS stated in a 2008 statement that MHT should start from the lowest effective dose [20]. Generally, 0.625 mg/day conjugated estrogen or its equivalent dose is called a standard dose, and half of the standard dose is called a low-dose hormone, such as 0.3 mg/day conjugated estrogen, 1 mg/day estradiol valerate, and 1 mg/day l7-beta-estradiol. About 1/4 of the standard dose is called an ultra-low dose, such as 0.5 mg/day estradiol valerate and 14 μg/day transdermal estrogen. The use of preparations of lower-than-standard doses can also greatly improve and maintain patients’ quality of life and is more conducive to reducing adverse drug reactions.

#### 4.3.2 Efficacy and safety of low-dose MHT

Hormone replacement therapy can significantly reduce menopausal symptoms associated with menopausal women, and guidelines for managing perimenopausal symptoms now recommend the lowest effective dose of estrogen. One study compared low-dose HRT (1 mg estradiol/2.5 mg dydrogesterone) and ultra-low dose HRT (0.5 mg estradiol/5 mg dydrogesterone) with placebo for 13 weeks, with all groups displaying positive therapeutic effects. The ultra-low dose group had significantly higher efficacy than that of the placebo group, and the incidence of vaginal bleeding was relatively low, which increased the users’ compliance and acceptance [21]. The Women’s HOPE study showed that low-dose hormonal components also improved related menopausal symptoms and provided some endometrial protection. The most unique therapeutic effect of MHT is that it can improve vaginal atrophy and related problems in women, especially dyspareunia. Its mechanism of action is to improve the maturation index of women’s vaginal cells, increase the number of vaginal wall cells, and enhance the local resistance of the vagina, thereby relieving vaginal discomfort and improving quality of life for women. The recommended best treatment plan is to choose low-dose hormones administered orally or vaginally. Relevant studies have shown that, even with topical treatment, the combined effect of lower doses of vaginal estrogen is better than that of higher doses. The effectiveness of low-dose hormone replacement therapy has been confirmed by a number of clinical studies. The shared conclusion of these studies is that, compared with standard-dose hormone replacement therapy, low-dose HRT can effectively relieve VMS and vulvovaginal symptoms in menopausal women, as well as prevent postmenopausal bone loss and reduce adverse effects. It can also improve the compliance of perimenopausal patients.

## 5 Non-hormonal drug options for perimenopausal syndrome

For perimenopausal syndrome, individualized treatment is always required, and the therapeutic effect and impact achieved for different individuals also vary greatly. For women who have no contraindications to hormone therapy and are willing to accept hormone therapy, hormone therapy can be recommended. However, for patients with contraindications to MHT or patients who are unwilling to receive hormone therapy, non-hormonal drug therapy is a good choice. Non-hormonal drugs include Chinese patent medicines such as Kuntai Capsules, Xiangshao Granules, and botanicals such as Black Cohosh.

**Figure 3:**
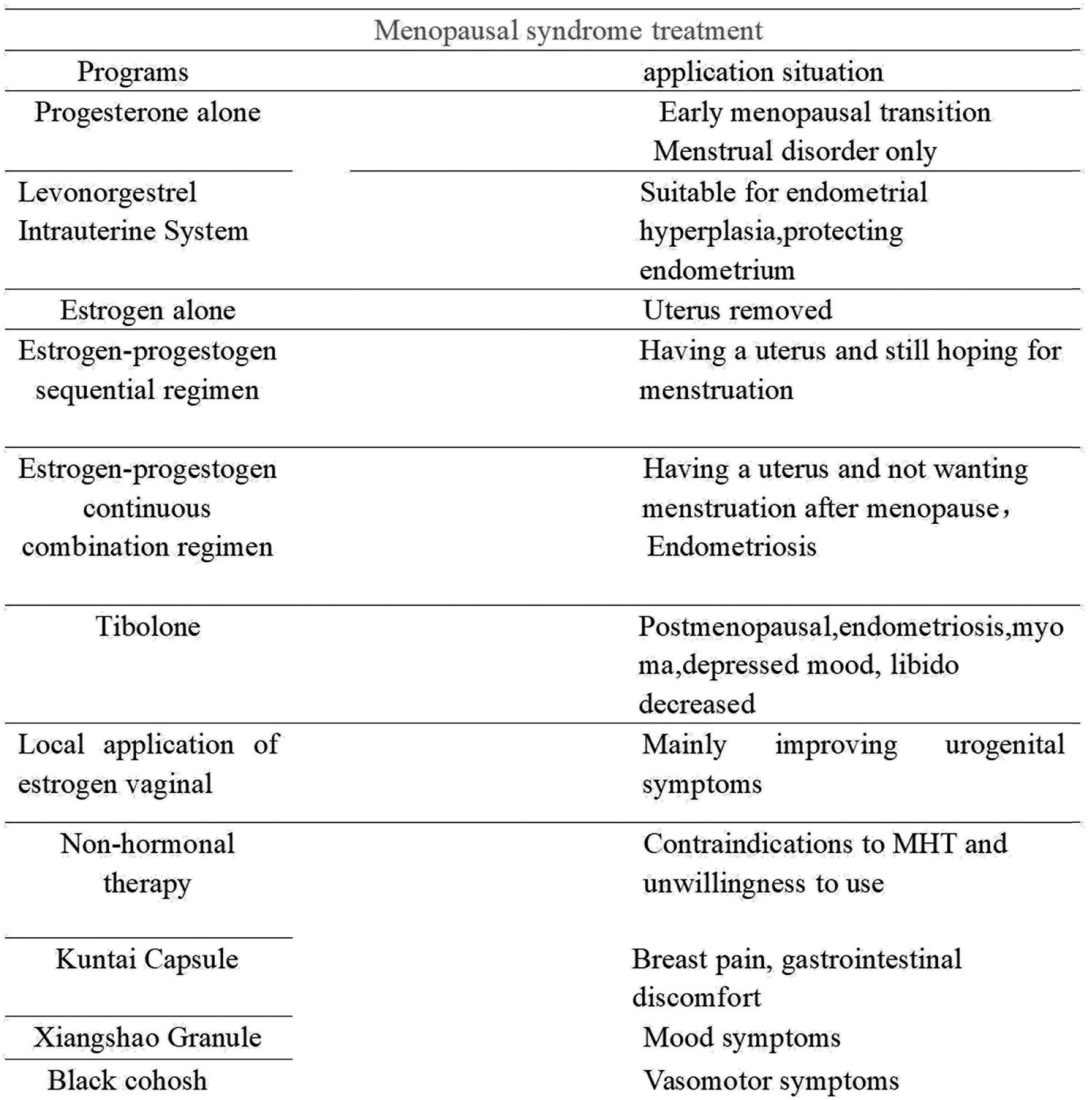
Treatment regimen selection.

**Figure 4:**
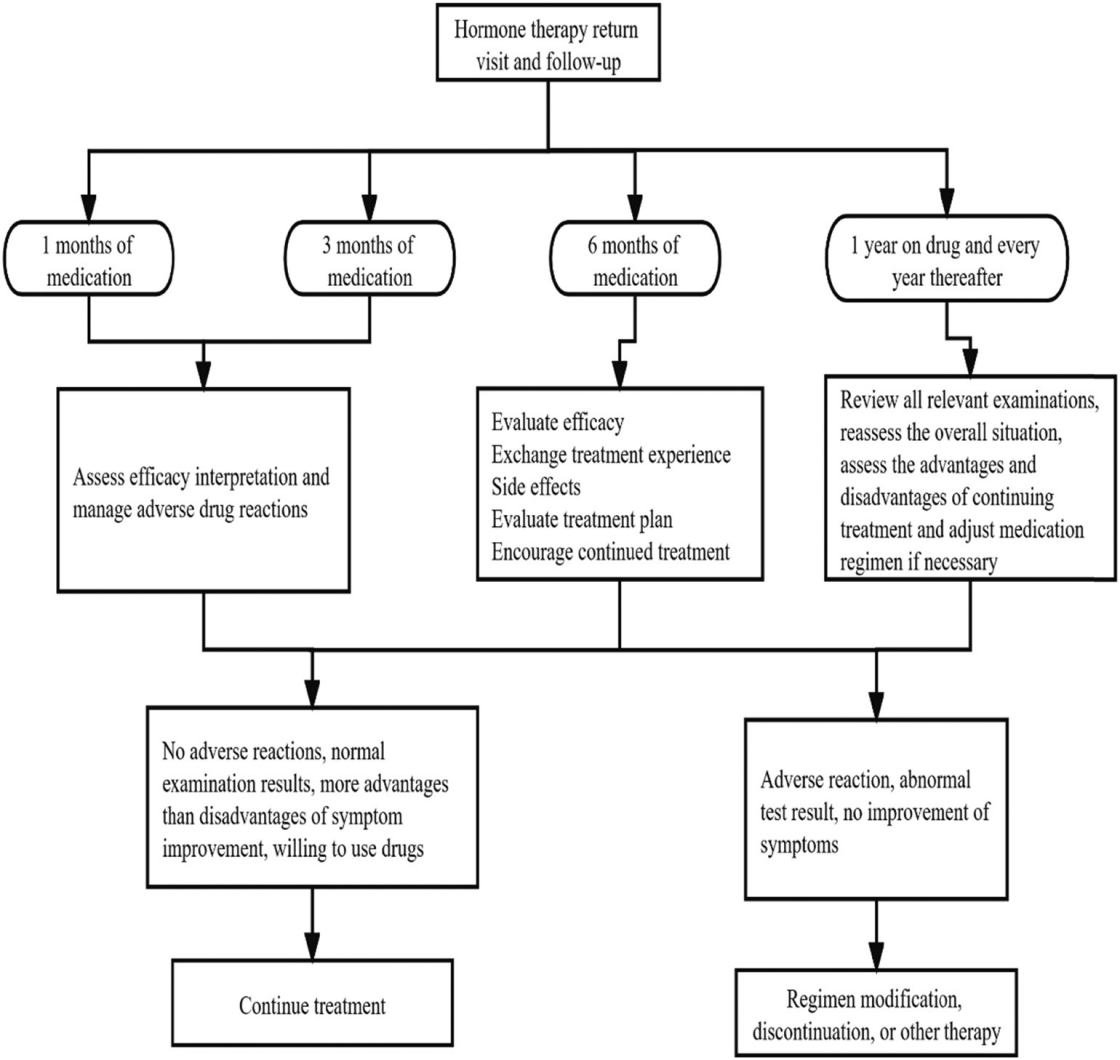
Re-examination and follow-up after treatment.

A meta-analysis showed that the incidence of adverse reactions to Chinese patent medicine Kuntai Capsule in the treatment of perimenopausal syndrome was lower than that of estradiol valerate, suggesting that Kuntai is safer in the treatment of menopausal symptoms and can reduce breast distending pain, vaginal bleeding, and other adverse reactions. Studies have shown that the Kupperman score, breast tenderness, gastrointestinal discomfort, and other symptoms after treatment with Kuntai Capsules were lower than those treated with hormone therapy, and the difference was statistically significant. Therefore, Kuntai Capsules can also solve the troubles caused by menopause and improve the symptoms of perimenopausal syndrome. Kuntai Capsules are an option for perimenopausal patients who cannot or are unwilling to use hormone therapy. A controlled clinical trial showed that the use of Xiangshao Granules can effectively improve mood disorders in menopausal women, and the Kupperman score decreased significantly after medication. Black Cohosh is a pure natural botanical medicine extracted from cohosh plants using modern technology. It is non-hormonal and has no hormone activity but can produce hormone-like effects. Most variants are used to relieve hot flashes and sweating and lower the frequency of VMS in menopause. Chinese guidelines on menopause management and menopause hormone therapy (2018) also believe that the above drugs are helpful for the improvement of menopausal symptoms (Figures 3 and 4).

## 6 Summary and outlook

As women age, the number of primordial follicles in their ovaries also decreases progressively. When the number of follicles drops to a very low level, women will experience menopause, and a series of symptoms associated with menopause are called perimenopausal syndrome. With the development and progress of society, people’s quality of life and life expectancy continue to improve, and the duration of menopause also increases for women. Therefore, it becomes a significant and challenging task to address menopause-related issues for women. The hormone therapy currently widely used can address 70–90% of perimenopausal symptoms. With research progressing, individualized and low-dose therapy is proposed. It is found that the lower dose of drugs taken by menopausal women can not only reduce patients’ economic burden but also effectively relieve menopause-related symptoms to improve the quality of life for women who are troubled by menopause. Furthermore, they can also reduce the adverse reactions caused by hormone therapy.

MHT is currently the main treatment for menopause-related symptoms, but it is not the only treatment. For patients with absolute contraindications or who do not wish to use hormonal therapy, general therapy and non-hormonal therapy can also reduce perimenopausal symptoms. The effects and risks of ultra-low-dose hormone therapy for patients who have passed the window period and have severe symptoms still need to be further explored. For patients who are reluctant to accept hormone therapy or have contraindications, the efficacy, safety, and individualized treatment plan of non-hormone therapy still need more conclusive data, and the safety and efficacy of long-term treatment with traditional Chinese medicines combined with hormone therapy also need further research and discussion.
